# Supplementary material for: Effectiveness of Booster Doses of the SARS-CoV-2 Inactivated Vaccine KCONVAC against the Mutant Strains
Source: Viruses. 2022 Sep 12;14(9):2016. doi: 10.3390/v14092016 (PMC9503905; doi:10.3390/v14092016)
Supplement: Supplementary file 1 [file viruses-14-02016-s001.zip › Table S1 Participant information of the inactivated SARS-CoV-2 vaccine cohort.pdf]

Table S1 Participant information of the inactivated SARS-CoV-2 vaccine cohort

ID	HLA-A2	Sex	Age
1	Yes	Male	50
2	No	Female	32
3	No	Male	24
4	No	Male	26
5	Yes	Female	24
6	No	Female	39
7	Yes	Male	24
8	Yes	Male	24
9	Yes	Male	24
10	No	Female	25
11	No	Female	24
12	No	Female	26
13	No	Male	22
14	Yes	Male	44
15	No	Male	31
16	Yes	Male	23
17	Yes	Male	33
18	Yes	Female	31
19	Yes	Female	25
20	No	Female	25
21	Yes	Male	31
22	No	Female	39
23	No	Female	32
24	No	Male	29
25	No	Male	33
26	Yes	Male	24
27	Yes	Male	31
28	No	Female	25
29	Yes	Male	30
30	No	Male	27
31	No	Female	24
32	Yes	Male	32
33	No	Male	43
34	Yes	Male	33
35	No	Male	28
36	No	Male	26
37	No	Male	24

38	No	Female	55
39	No	Male	23
40	Yes	Male	32
41	No	Male	29
42	Yes	Male	31
43	Yes	Male	24
