# Supplementary material for: Effectiveness of Booster Doses of the SARS-CoV-2 Inactivated Vaccine KCONVAC against the Mutant Strains
Source: Viruses. 2022 Sep 12;14(9):2016. doi: 10.3390/v14092016 (PMC9503905; doi:10.3390/v14092016)
Supplement: Supplementary file 1 [file viruses-14-02016-s001.zip › Table S2 Participant information of convalescent COVID-19 patients.pdf]

Table S2 Participant information of convalescent COVID-19 patients

Donor code	HLA-A2	Sex	Age	Variant Strain
# 1	Yes	Female	39	B.1.617.2 (Delta)
# 2	No	Female	74	B.1.617.2 (Delta)
# 3	No	Male	76	B.1.617.2 (Delta)
# 4	No	Female	38	B.1.617.2 (Delta)
# 5	No	Female	53	B.1.617.2 (Delta)
# 6	Yes	Male	45	B.1.617.2 (Delta)
# 7	Yes	Male	60	B.1.617.2 (Delta)
# 8	No	Female	69	B.1.617.2 (Delta)
# 9	No	Female	42	B.1.617.2 (Delta)
# 10	No	Female	74	B.1.617.2 (Delta)
# 11	No	Male	48	B.1.617.2 (Delta)
# 12	No	Male	51	B.1.617.2 (Delta)
# 13	Yes	Female	40	B.1.617.2 (Delta)
# 14	No	Male	59	B.1.617.2 (Delta)
# 15	Yes	Female	48	B.1.617.2 (Delta)
# 16	No	Male	64	B.1.617.2 (Delta)
# 17	No	Female	53	B.1.617.2 (Delta)
# 18	Yes	Male	11	B.1.617.2 (Delta)
# 19	No	Female	62	B.1.617.2 (Delta)
# 20	No	Female	69	B.1.617.2 (Delta)
